# Ethanol combined with energy drinks: Two decades of research in rodents

**DOI:** 10.3389/fnbeh.2022.1100608

**Published:** 2023-03-02

**Authors:** Beatriz Nunes Petribu, Karina Possa Abrahao, Maria Lucia Oliveira Souza-Formigoni

**Affiliations:** Departamento de Psicobiologia, Escola Paulista de Medicina, Universidade Federal de São Paulo, São Paulo, São Paulo, Brazil

**Keywords:** ethanol, rodents, locomotor activity, consumption, AmED, energy drinks (EDs)

## Abstract

Many studies raised concerns on alcoholic beverages consumption mixed with energy drinks (AmED), which can induce higher rates of binge drinking and earlier development of alcohol use disorders. After 20 years of research, few studies with laboratory animals have focused on the effects of this mixture and the neurobiological and pharmacological mechanisms underlying them. We found 16 articles on AmED administration to rodents evaluating its effects on voluntary consumption, locomotion, anxiety-like behavior, memory, influence on the onset time of seizures, biochemical and neurochemical measures. Some of these studies indicated energy drinks (ED) can alter the pattern of use and motivation to consume ethanol (EtOH); increase the expression of sensitization to EtOH stimulant effect and the proportion of sensitized mice; decrease the aversiveness of high concentrations of EtOH, among other effects. In addition AmED hastens the loss of righting reflex and its effects on memory are controversial. After acute administration no difference was found in blood ethanol concentration (BEC) of rodents which received EtOH with or without ED, but after 60 days of treatment, AmED group had lower BEC levels than EtOH group. Data on biochemical and neurochemical parameters after AmED are not consistent. Although the AmED group presented higher glucose levels than the EtOH group when drugs were administered by *gavage*, this was not observed in a self-administration protocol. AmED may induce higher kidney damage, higher levels of plasma urea, uric acid and creatinine when compared to EtOH. Chronic consumption of AmED causes an inflammatory response and oxidative stress, which may induce cell death in the cortex and hypothalamus of adult rats. These controversial results show that AmED diverse effects depend on sex, age and lineage of the animals, duration of the treatment and route of administration. Further research is necessary to evaluate the mechanisms underlying AmED biological effects.

## 1. Introduction

Energy Drinks (ED) consumption has increased in the last few years. According to the website of the manufacturer of the first energy drink (Red Bull™), launched in Austria in 1987, almost 10 billion cans of Red Bull were sold worldwide in 2021, representing an increase of 24.3% against 2020. These drinks are usually composed of sucrose or glucose, taurine, caffeine, gluconolactone, inositol, niacin, panthenol, vitamins (B2/B6/B12), citric acid, caramel coloring, artificial flavoring, and sparkling water. Despite the concerns about ED drinking behavior *per se*, many studies have alerted that alcoholic beverages mixed with energy drinks (AmED) can also be considered a growing public health issue (Kaur et al., 2022). The increased consumption of AmED has been attributable to the fact it makes the consumer feel less sedated, improves the taste of alcohol and allows drinking for a longer period (Marczinski, 2011; Johnson et al., 2016). O’Brien et al. (2008) reported a strong association between the use of AmED with increased heavy alcohol episodic drinking (binge drinking) and twice as many episodes of weekly drunkenness. When compared to alcohol drinking, AmED consumption has also been associated with higher rates of risky sexual behavior, impaired driving, and risk to develop alcohol use disorders (Marczinski and Fillmore, 2014). In previous studies (Ferreira et al., 2004a,b, 2006) our research group evaluated the patterns of use, the physical and the neuropsychological effects of AmED in humans. However, after almost two decades of research in this area, there is a paucity of studies on the effects of AmED in animal models. The aims of this review are to provide a summary of the results observed in studies using animal models conducted in the last 20 years and to evaluate the interaction between EtOH and energy drinks according to specific behavioral models and physiological measures.

## 2. Methods

We used the following keywords and MeSH terms to find the research articles of interest: (alcohol OR ethanol) AND (“energy drinks” OR “energy drink”) AND (mice OR rats OR rodents) in the site of EBSCO*host* including the follow databases: Academic Search Premier, CAPES FSTA Full Text Collection, CINAHL with Full Text, Computers and Applied Sciences Complete, Dentistry and Oral Sciences Source, Food Science Source, FSTA–Food Science and Technology Abstracts, Information Science and Technology Abstracts (ISTA), Library, Information Science and Technology Abstracts with Full Text, MEDLINE Complete, MLA Directory of Periodicals, MLA International Bibliography, RILM Abstracts of Music Literature, RIPM–Retrospective Index to Music Periodicals, SocINDEX with Full Text and SPORT Discuss with Full Text. As a result, we found 57 articles, but most of them evaluated the effects of the mixture of EtOH and caffeine or any other ED component instead of the original energy drinks. Considering the focus of this review was to analyze studies in rodents exposed to EtOH mixed with commercially available energy drinks, studies with only the main components, such as caffeine or taurine, were excluded, resulting in 18 articles. Two were excluded because they had only the abstract available in English and the full text in Russian. The remaining 16 articles were analyzed by two of the authors (MLOSF and BNP) and included in this review.

## 3. Results

Supplementary Table 1 shows the references, methods, and main findings of the 16 articles included in this review. Some of them focused only on specific indicators but others combined different behaviors and physiological measures. The results are presented according to the behavior or physiological measure analyzed.

### 3.1. Behavioral studies

#### 3.1.1. Voluntary consumption

Three studies investigated the effects of ED on voluntary EtOH consumption. There was no difference in EtOH drinking by C57BL/6 male mice previously exposed to a voluntary access to ED during adolescence (10 days) or during adolescence + adulthood (4 weeks) (Robins et al., 2016). When exposure is longer, from adolescence to adulthood, the effects can be different. After a 10- week intermittent chronic self-administration of AmED, female rats show higher motivation in a progressive ratio task than animals pre-treated with ED only or daily AmED (Williams et al., 2022). EtOH itself can be aversive to rats, but it seems that ED can decrease the aversiveness of high concentrations of EtOH. Indeed, in a self-administration protocol all doses of EtOH were aversive compared to sucrose, but a higher EtOH dose was necessary to decrease the number of responses to AmED in an operant chamber (Roldán et al., 2017). When ED is combined with 20% EtOH, AmED rats drink more than those pressing for 20% EtOH + sucrose (Roldán et al., 2017). It is interesting to note that no study evaluated how previous exposure to EtOH can change ED or AmED drinking behavior, an important topic to be covered by future research.

#### 3.1.2. Motor behavior tests

There is evidence linking the reinforcing and locomotor-stimulating effects of drugs of abuse. Addictive drugs have psychomotor stimulant properties whose underlying biological mechanisms are probably the same, or have common elements with the biological mechanism of the reinforcing properties of those drugs (Wise and Bozarth, 1987). That’s why there is a big concern about the psychostimulant effects of ED and AmED. Out of the 16 articles selected, half of them provided measures of locomotor activity, using activity boxes equipped with photodetectors or open field apparatus. A pioneering study, using Swiss mice, showed that ED induced a stimulant effect *per se* on locomotion and its administration, in a dose equivalent to three cans of energy drink, reduced the effect of a depressant dose of EtOH to the levels of the control group (Ferreira et al., 2004b). However, in another study, an acute stimulant effect of AmED was observed during the initial 5 min of the test (Takahashi et al., 2015). In mice, the stimulant effect induced by AmED was observed only 1 h after a single *gavage* administration (Krahe et al., 2017). If the locomotor measures are taken 12 h after an acute treatment of AmED, mice show lower locomotor activity when compared to those treated with ED or EtOH (Asorey et al., 2018). It is important to point out that only Asorey et al. (2018) controlled for the caloric component of a *gavage* administration of ED, using sucrose in the control group, while all other studies used water. This may be an important point to be considered in future studies on the acute interaction effects of AmED.

It is well documented, for female Swiss mice, that the repeated administration of EtOH can lead to a progressive increase of the locomotor stimulant effect, a phenomenon known as behavioral sensitization (Souza-Formigoni et al., 1999). Nevertheless, we did not find studies on the effects of AmED on behavioral sensitization in male mice. Ferreira et al. (2013) assessed how ED affects the expression of EtOH sensitization in female mice. The administration of AmED triggered the expression of EtOH sensitization even in animals previously classified as “low-sensitized” (those that did not show sensitization to EtOH alone). This result suggests AmED could potentiate the stimulant effects of EtOH in female mice (Ferreira et al., 2013). In another study, no difference was observed in locomotion during 5 min of test among male and female Wistar rats treated three time a week for 4 weeks with EtOH, ED, AmED, caffeine, EtOH + caffeine, taurine, EtOH + taurine, caffeine + taurine, and EtOH + caffeine + taurine (Costa-Valle et al., 2022). Although a chronic operant self-administration of ED for 15 days increased the locomotion of male rats, a previous history of ED self-administration did not change EtOH seeking or EtOH-induced stimulant locomotor effects (Roldán et al., 2017). Williams et al. (2022) showed that although ED did not induce behavioral sensitization in female rats, 10 weeks of intermittent AmED self-administration increased locomotion, indicating development of behavioral sensitization only after a long exposure to EtOH and ED combination.

It is possible to investigate motor coordination using different types of tests. In the rotarod test, no difference was observed one or 3 h after a single oral administration of the AmED or EtOH alone (Krahe et al., 2017). However, 12 h after AmED treatment, mice showed higher impairment of the motor coordination than those treated with EtOH, when using the tightrope and the hang wire tests (Asorey et al., 2018). Swiss male and female mice need less oral *gavage* administration of AmED to lose their righting reflex than mice receiving EtOH only. The effects of ataxic doses of EtOH vary depending on the time of substance elimination from the body (Krahe et al., 2017).

#### 3.1.3. Anxiety-like behavior

There is an interaction between anxiety and EtOH consumption. While higher anxiety behavior can lead to EtOH drinking in pursuit of its anxiolytic effects (Wilson et al., 2004), chronic EtOH administration leads to anxiety-like behaviors during its withdrawal (Bloch et al., 2022). The effects of AmED on anxiety vary according to treatment regimen protocols. Four studies were found on this theme. AmED induced an anxiogenic effect 1 h after a single oral *gavage* administration, although there was no difference when compared with EtOH alone (Krahe et al., 2017). In agreement with this and using male Swiss mice, Asorey et al. (2018) found no difference in anxiety-like behavior induced by EtOH or AmED. In both studies, drugs were acutely administered, but while in the first study the animals were tested 1 h after administration, in the second one they were tested 6, 12, and 18 h afterward. Chronic exposure to AmED or ED may lead to different changes in anxiety-like behaviors. Although there was no difference between EtOH alone and AmED in anxiety-like behavior of male and female Wistar rats treated 3 times a week for 4 weeks (Costa-Valle et al., 2022), an anxiolytic effect was observed in Sprague–Dawley female rats exposed to an intermittent access to AmED, for 10 weeks. This effect was not observed in rats exposed to ED (Williams et al., 2022), but in that study they were not compared with animals which received only EtOH. In addition to the different periods of administration, the chronic studies worked with female rats, while most of the acute studies used male Swiss mice. The different results found between these two articles can also be due to the different lineages of rats and time of treatment. Other issues can be explored in this topic which is of great importance considering the relevant relationship between anxiety and drug abuse.

#### 3.1.4. Memory tests

Three studies focused on the effects of chronic exposure to EtOH and/or ED on memory tasks. To study the short-term memory function in male Wistar rats, an object recognition task and a social discrimination test were conducted 2 and 3 days after 6 days of drugs administration, respectively. Animals treated with EtOH alone presented discrimination deficits to a new object or to a novel social interaction, an effect that was not reverted by AmED previous administration (Takahashi et al., 2015). Tests on object recognition conducted in male Wistar rats showed long-term memory improvement in the group treated with ED components (caffeine and taurine) but not in the ED group, which could be related to other components present in the energy drink (Valle et al., 2018). In a recent study of the same authors, rats were treated with a binge drinking protocol 3 days a week over 4 weeks, which included oral gavage of water, energy drink, caffeine, taurine, and their combinations with EtOH. Although EtOH alone reduced their performance in the memory test, when caffeine and taurine were administered in combination with EtOH, this effect did not appear, and long-term memory was maintained (Costa-Valle et al., 2022). Using Sprague–Dawley females, no effect of ED or AmED was detected in the recognition of a novel position of the object, after 10 weeks of access to drugs (Williams et al., 2022). These results indicate that the effect of the combination of the drugs on short-term memory tasks may be time dependent. In long-term memory, in a Conditioned Place Preference test, 1 to 3 weeks after the last drug administration, all groups of animals (ED, EtOH, or AmED) showed a side preference after the conditioning phase, but this behavior was stronger in the AmED group (Takahashi et al., 2015), indicating that ED can potentialize a long-term environmental conditioning memory. This diversity in results should be the focus of future studies.

#### 3.1.5. Seizures

Another recent study analyzed the effects of ED, EtOH and AmED in rats treated with pentylenetetrazol (PTZ), a seizure inductor. AmED and EtOH shortened onset time of the seizures and the severity of PTZ-induced seizures (Gözler and Uzbay, 2022).

### 3.2. Physiological parameters

#### 3.2.1. Blood ethanol concentration (BEC)

The behavioral effects of AmED might be associated with an effect of ED on EtOH metabolization. However, in acute treatment, there was no difference in BEC between EtOH or AmED groups, 30 min after the administration (Ferreira et al., 2004b). This pattern was also observed in the BEC levels of male and female mice 1 h after oral gavage of EtOH or AmED (Krahe et al., 2017). Additionally, there was no effect of AmED on EtOH metabolization 60, 180, and 360 min after the drug administration (Asorey et al., 2018). However, metabolization can be affected after chronic administration of the drugs. Male mice treated with AmED and EtOH for 5 days did not show a difference in BEC 30 min after administration of the drugs (Takechi et al., 2021). On the other hand, if the blood is collected immediately after the last session of a 15-day chronic self-administration protocol with ED followed by a 20-day self-administration protocol with AmED, BEC is significantly higher than those measured in animals from the EtOH + sucrose group (Roldán et al., 2017). A different result was seen after 60 days of treatment. In this case, although authors did not report a statistical difference, male rats under a chronic EtOH treatment presented BEC of 67 mg/dL, while those receiving AmED presented 30 mg/dl (Díaz et al., 2016). An important concern regarding the latter study is that the authors did not mention when the blood sample was collected. Consequently, this result could be associated with an effect of the time or a faster depuration of EtOH when ED is onboard. The type of administration may impact the BECs measures considering that animals under a self-administration protocol can have a variable amount of EtOH in their bodies, while animals receiving drug by *gavage* have an experimenter-controlled dose of EtOH.

#### 3.2.2. Biochemical and neurochemical measures

Regarding biochemical and neurochemical measures, there is only one article about acute AmED treatment, and seven articles related to chronic AmED treatment. Twenty-four hours after an acute treatment there was no difference in urea, creatinine, alanine aminotransferase (ALT), aspartate aminotransferase (AST), γ-glutamyltranspeptidase (γ-GT), alkaline phosphatase (ALP), and lactate dehydrogenase (LDH) levels of male rats treated with AmED or EtOH (Costa-Valle et al., 2018). Despite this, when animals were chronically treated with AmED for 30 days, significantly higher levels of total white blood cell counts (TWBC) and plasma calcium were found in the AmED groups when compared with the control one, but there was no difference between AmED and ED groups. The groups treated with AmED had significantly higher levels of aspartate transaminases (AST), ALP and total bilirubin in comparison with the control group (Ugwuja, 2014). Another study showed that after 30 days of treatment, there was no difference between EtOH and AmED groups in AST activity in serum and in myocardium. However, the EtOH group had less ALT activity in serum than controls. Higher ALT activity in myocardium was found only in the ED group, compared with control, with no difference between EtOH and AmED groups (Munteanu et al., 2018).

The AmED group had a higher glucose level than the group of EtOH alone, although there was no difference between them in the glycogen levels of male Wistar rats treated by *gavage*. Interestingly, only ED presented higher levels of glycogen, showing that the mixture with EtOH can change this parameter (Munteanu et al., 2018). In contrast, when drugs were delivered in an operant self-administration paradigm there was no difference between AmED and EtOH alone regarding glucose levels measured before (basal) and after (5, 30, and 90 min) the self-administration session (Roldán et al., 2017). In this same paradigm, there was no difference between these two groups in relation to insulin and corticosterone levels measured *in vivo* immediately after the 30-min session and immediately after animals death (Roldán et al., 2017). As regards the impact of the mixture on cholesterol levels, there was no difference between animals treated with AmED and EtOH (Munteanu et al., 2018). Rats treated with AmED for 30 days had higher levels of plasma triglyceride than those of the group treated with ED only or the control group (Ugwuja, 2014). However, regarding protein concentration, animals treated with EtOH alone had higher levels than the control group (Munteanu et al., 2018).

As to the nephrotoxicity, the group acutely treated with AmED had higher N-acetyl-β-D-glucosaminidase (NAG) activity compared with all other groups, evidencing kidney damage (Costa-Valle et al., 2018). After a chronic treatment, an increase in plasma urea, uric acid and creatinine observed in the groups with high doses of ED and AmED was considered by the authors a possible indicator of kidney malfunctioning (Ugwuja, 2014). It is important to point out that in this latter study they did not administer EtOH alone. These results suggest that AmED can cause kidney damage both acutely and chronically.

Regarding oxidative stress, no alterations in plasma malondialdehyde (MDA) or thiobarbituric acid reactive substances (TBARS) levels were found between ED or AmED after an acute treatment (Costa-Valle et al., 2018). However, after 14 days of treatment, MDA levels in the AmED group were higher than in the EtOH alone in a dose-dependent manner. No difference was observed in SOD, CAT, and GSH-Px activity between animals treated with EtOH alone or mixed with ED (Reis et al., 2017). After 60 days of treatment, the AmED group presented higher lipid peroxidation and formation of reactive oxygen species than the EtOH alone (Díaz et al., 2016). C57BL/6 male mice treated with EtOH alone had more blood–brain barrier dysfunction in the hippocampal formation compared to the controls and AmED groups. No difference was found in the cortex. The AmED group presented significantly less astroglial expression, less GFAP marking in the hippocampus and a reduced expression of Iba-1 compared to EtOH alone after 5 days of treatment (Takechi et al., 2021). In contrast, after 60 days of treatment, the AmED had increased immunoreactivity to GFAP in comparison with EtOH and water control groups. AmED also increased the concentration of IL-1β and TNF-α levels compared with EtOH alone in male Wistar rats. No difference was found in NO2 levels of AmED and EtOH (Díaz et al., 2016).

A very recent paper was the first to investigate the mixture of ED and EtOH on reproductive parameters. In females, the estrous cycle regularity, the relative mass of the ovaries, and the quantity and quality of oocytes was evaluated. In males, the relative mass of testicle, right and left epididymis, seminal vesicle, prostate, the number of spermatids and sperm, morphological assessment of sperm, and testosterone level were evaluated. However, no difference was found between groups in reproductive parameters for both sexes (Costa-Valle et al., 2022).

## 4. Discussion

There are few studies with animals focusing on the effects of the mixture of EtOH and ED on behavioral and physiological variables. The use of animal models can contribute to better understand the impacts of EtOH and ED on behavior and also to investigating the neurobiological and pharmacological mechanisms underlying them. In this review, we synthesized the main findings available so far, considering the treatment regimen and the timing of each measure. Few articles used the same protocol which limits the comparison among their results. In general, the administration of AmED, acutely or chronically, increased the voluntary consumption of EtOH, as well as the motivation to consume it, stimulates locomotion in a dose-dependent manner and worsens the motor performance. AmED administration decreased anxiety in a time-dependent manner but there is a diversity of data on its effects on memory. Acute treatment with AmED did not change the metabolization of EtOH, but chronically, AmED induced changes on BEC levels depending on the protocol employed. A similar diversity was found regarding metabolic dysfunction. AmED increased the number of pro-inflammatory cytokines, induced oxidative stress and lipid peroxidase, but these results also vary according to the paradigm utilized. In summary, AmED causes different consequences according to the doses of EtOH and ED and the lineage, age and sex of animals. These results agree with clinical studies.

The importance of using animal models in the study of the interaction between EtOH and ED is given by the possibility of controlling many variables in these studies. This is crucial to understand some of the mechanisms involved, as well as their consequences. To compare results from different studies it is recommended the use of standardized protocols. Future studies are necessary to shed light into the real impact of the popular mix of alcoholic beverages and energy drinks. If some of the results observed in studies with rodents are similar in human beings, it would be important to alert users and stakeholders on its possible deleterious consequences. For instance, the possibility of AmED increasing the reinforcing effects of alcoholic beverages could induce more people to use it and to be more prone to develop risk patterns of use, such as binge drinking and alcohol dependence.

## Author contributions

MLOSF and KPA contributed to the conception and design of the study. BNP organized the database and with MLOSF reads and selected the manuscript to be included in the review. BNP wrote the first draft of the manuscript, revised by KPA and MLOSF. All authors contributed to the final version of the manuscript, read, and approved the submitted version.
