# Supplementary material for: Ethanol combined with energy drinks: Two decades of research in rodents
Source: Front Behav Neurosci. 2023 Mar 2;16:1100608. doi: 10.3389/fnbeh.2022.1100608 (PMC10017554; doi:10.3389/fnbeh.2022.1100608)
Supplement: Supplementary file 1 [file Table_1.pdf]

Table 1 – Main characteristics of the studies in rodents on the effects of Alcohol mixed with Energy Drinks (AmED) published from 2004 to 2022 (by order of publication).

	Lineage	Sex	Groups	Animals per Group	Route and period of Administration	Behavioral Tests	Physiological Parameters	Results	Authors
Mice	Swiss	Male	Control, progressive EtOH (0.5g/kg, 1.0g/kg, 1.5g/kg, 2.5g/kg), ED (3.57 ml/kg, 10.71 ml/kg, 17.85ml/kg) and AmED (EtOH doses+ ED 10.71 ml/kg)	16 to 20	Gavage, Acute	Locomotor activity (activity boxes/45 min)	Blood EtOH Concentration (BEC) 30 min after drug administration	Locomotion: ED > control; AmED > EtOH 2,5g/kg = Control;<br>BEC: AmED = EtOH	Ferreira et al., 2004
Mice	Swiss	Female	Control, EtOH (2.4 g /kg), AmED (2.4 g /kg EtOH + 10.71 ml/kg ED)	23-30	I.P. or Gavage, Chronic (21 days) and acute	Locomotor activity (activity boxes) 15 min/ immediately after the administration	-	Most of the mice treated with EtOH (21 days) developed sensitization to their stimulant effect. AmED increased the intensity of the expression of sensitization and proportion of mice which presented high sensitization	Ferreira et al., 2013
Rat	Albino	Male	low dose ED (3.75ml/Kg), high dose ED (7.5ml/Kg), low dose AmED (3.75ml/Kg ED + 1.0g/Kg EtOH) and high dose AmED (7.5 ml/Kg ED + 2.0 g/Kg EtOH). Absence of EtOH only group.	4	Not specified, Chronic (30 days)	-	Measure of packed cell volume (PCV), haemoglobin concentration (HBC) and total white blood cell counts (TWBC), plasma calcium, sodium, potassium, albumin, uric acid, plasma urea, creatinine, total cholesterol, triglyceride concentrations, plasma alanine transaminase (ALT), aspartate transminases (AST), alkaline phosphatase (ALP) and bilirubin.	Higher levels of TWBC and plasma calcium in AmED compared with control, but no difference regarding ED.<br>Higher plasma urea, uric acid and creatinine observed in the groups with high doses of ED and AmED than in control.<br>High dose AmED: higher levels of aspartate transaminases (AST), alkaline phosphatase (ALP) and total bilirubin than control group, no difference regarding ED group.<br>The plasma triglyceride levels in AmED were higher than ED only and control groups.	Ugwuja, 2014
Rat	Wistar	Male	Control, EtOH (1 or 3,4 g/kg), ED (10,71 ml/kg) and AmED	10 to 13	Gavage, Chronic (6 days)	Locomotor activity (activity boxes/ 30 min, 15 min after administration in day 1) Memory: object recognition test / 2 days after the administration for 2 days), a social discrimination test (3 days after the last administration) and Conditioned Place Preference (13 days / between 1 and 3 weeks after the administration)	-	Locomotor activity: AmED > EtOH alone (first 5 min).<br>AmED and EtOH equally decreased the perception of novel and familiar objects and animals. CPP test: the 3 groups presented more time in the EtOH-paired compartment (post-condition vs pre-condition) AmED preference> EtOH	Takahashi et al., 2015

Rat	Wistar	Male	Control, ED (7.5 mL/kg), EtOH (2.5 g/kg) and AmED	8	Gavage, Chronic (60 days)	-	BEC (last day of treatment). immunoreactivity to GFAP, concentration of IL-1β and TNF-α, Nitric Oxide (NO), Lipid Peroxidation and Reactive Oxygen Species	BEC of EtOH alone was higher than AmED. AmED increased reactive gliosis, IL-1β, TNF-α, iNOS, reactive oxygen species, lipid peroxidation, and nitric oxide, in the cortex and hypoccampus. In the same regions immunoreactivity to caspase-3 and a decrease of synaptophysin were detected, different than EtOH. AmED presented higher lipid peroxidation and formation of reactive oxygen species than EtOH alone.	Díaz et al., 2016
Mice	C57BL/6 BALB/c	Male	EtOH (1%, 3%, 6%, 12% e 20% v/v), ED (sucrose (120g/L) + caffeine (326 mg/mL or 550 mg/mL)) and sucrose + quinine (1.75mM)	3 to 6	Two Bottle Choice, Chronic (10 days only ED for 24h and 16 days only EtOH 4h/day); Chronic (ED alone 4h/day during 5 days for 4 weeks and EtOH alone for the same time)	Free access self-administration ED and water (24h for 10 days, free access) and after that, access to EtOH (4h/day for 16 days). In the limited access phase, animals self-administered water and/or ED (4h/day for 5 weeks) and after EtOH (4h/day for 20 days)	-	Consumption of EtOH (limited access or free access paradigm): EtOH = AmED	Robins et al., 2016
Mice	Swiss	Male and Female	Control, EtOH (4g/kg), ED (8ml/kg) and AmED	11 to 48	Gavage, Acute	Locomotor activity (open field/5 min) Loss of Righting Reflex Rotarod (2 sessions)	-	Locomotion: AmED > EtOH > controls. Loss of righting reflex: AmED sooner than EtOH alone. Rotarod: impaired motor coordination - AmED = EtOH alone	Krahe et al., 2017
Rat	Sprague-Dawley	Male	Control, low-dose ED (3,5 g/kg), high-dose ED (7,0 g/kg), low-dose AmED (1,0 g/kg EtOH +low dose ED) and high-dose AmED (1,0 g/kg EtOH + hifgh dose ED)	7	Gavage, Chronic (14 days)	-	Lipid peroxidation, MDA levels, oxidative stress (SOD, CAT, and GSH-Px activity in liver and brain tissues) (1 day after the last administration)	No difference between EtOH alone and AmED in SOD activity, CAT activity levels, GSH-Px activity. MDA levels: AmED > EtOH alone in an dose-dependent manner	Reis et al., 2017
Rat	Wistar	Male	ED (20 to 100%), Sucrose (2.2 to 11%), EtOH (3, 6, 10, 15 e 20%) and saccharin (0.005 %)	7 to 30	Operant self-administration, Chronic (15 days with only ED and 20 days with only EtOH)	Locomotor activity (activity boxes / 30 min, immediately after last administration) of operant self-administration of ED and EtOH. Animals had also past for the relapse-like drinking situation known as the alcohol deprivation effect, which last 7 day. They monitored alcohol relapse for 5 days and 10 more additional days for returning to their baseline.	Blood glucose before and after (5, 30 and 90 minutes) the self-administration session. Insulin, BEC and corticosterone levels (immediately after the final operant session of EtOH).	Consumption: In low concentration of EtOH (3% and 6%) EtOH group > AmED but in higher EtOH concentrations (15% and 20%) AmED group > EtOH. The number of active lever responses for EtOh 20%: AmED > EtOH alone. No difference in the locomotor activity, glucose, insulin and corticosterone levels between EtOH alone and AmED. BEC in self-administered protocol: AmED > EtOH alone. After abstinence: AmED group > active lever responses than EtOH alone.	Roldán et al., 2017

Mice	Swiss	Male	Saline + Sucrose (18ml/kg), Sucrose + EtOH (3,8 g/kg), Saline + ED (18ml/kg) and AmED	12 to 15	Gavage and i.p., Acute	Locomotor activity (open field/ 5 min). Anxiety like-behavior (open field/ 5 min and elevated plus maze/ 5 min). Tightrope test (horizontal rope/ 60 s). Hanging wire test (60s)	BEC (60, 180 or 360 minutes after administration)	Locomotion: AmED < EtOH + sucrose group, 12h after treatment. Anxiety-like behavior: EtOH = AmED Motor coordination: AmED < EtOH + sucrose both in tightrope and hangwire test, 12h after treatment. BEC: EtOH + Sucrose = AmED groups at 60, 180 and 360 min after administration	Asorey et al., 2018
Rat	Wistar	Male	Control, EtOH (2g/kg), ED (10 ml/kg), caffeine (3,2 mg/kg) + taurine (40 mg/kg), AmED and caffeine + taurine + EtOH	5	Gavage, Acute	-	Measures of urea, creatinine, alanine aminotransferase (ALT), aspartate aminotransferase (AST), γ-glutamyltranspeptidase (γ-GT), alkaline phosphatase (ALP), lactate dehydrogenase (LDH) levels, oxidative stress in tissue, and blood and nephrotoxicity (24h after the administration)	No difference between groups in urea, creatinine, ALT, AST, γ-GT, ALP, LDH and thiol levels. Significant increase (76%) in NAG activity in the urine of AmED, evidencing kidney damage. No diference in TBARS between AmED and EtOH, although ED or caffeine + taurine alone presented higher levels of TBARS in kidney. The AmED group had an increased urinary NAG activity compared with all other groups.	Costa-Valle et al., 2018
Rat	Wistar	Male	Control, EtOH (4,86mg/kg), ED (15ml/kg) and AmED	7	Gavage, Chronic (30 days)	-	Total glucose concentration, Glycogen concentration, Total cholesterol, Total protein concentration, AST and ALT activities and analyses of myocardium specimens (immeadiately after the test in the last day of administration)	After 30 days of administration AmED and ED increase heart glucose and glycogen levels and decreased total cholesterol. ALT and AST activities and total protein concentration increased in all groups. AmED group presented ultrastructural alterations indicators of onset of alcoholic cardiomyopathy	Munteanu et al., 2018
Mice	C57BL/6J	Male	Control, EtOH (37.5% alcohol/volume), ED (60%) or AmED (40:60)	5	Gavage, Chronic (5 days)	-	BEC, the integrity of blood-brain barrier (IgG), neuroinflammation (detection of activate astrocytes), microglia (GFAP and Iba-1) and serum cytokine (30 min after the administration)	BEC: no difference among groups that received EtOH. EtOH alone presented blood–brain barrier dysfunction and increased hippocampal GFAP, while the mixture with ED showed a protective effect. No difference between EtOH and AmED in Iba-1 levels.	Takechi et al., 2021

Rat	Wistar	Male and female	Control, EtOH (2 g/kg), ED (10 ml/kg), AmED, caffeine (3.2 mg/kg), EtOH + caffeine, taurine (40 mg/kg), EtOH + taurine, caffeine + taurine and EtOH + caffeine + taurine	10 and 5	Gavage, Chronic (3 days/week for 4 weeks)	Locomotor activity and anxiety (open field / 5 min). Memory: object recognition test (short memory/ 5 min and long-term memory / 5 min)	In females: estrous cycle regularity, relative mass of the ovaries, quantity and quality of oocytes. In males: relative mass of testicle, right and left epididymis, seminal vesicle, and prostate and the number of spermatids and sperm, morphological assessment of sperm, and testosterone level	No difference between EtOH and AmED groups in anxiety, memory and reproductive profiles in female and male rats. AmED increased locomotor activity in females and long-term memory in males.	Costa-Valle et al., 2022
Rat	Wistar	Female	PTZ (60 mg/kg) + Saline, PTZ + Caffeine (3,43 mg/kg), PTZ + ED (10,71 ml/kg), PTZ + EtOH (3,4 g/kg) and PTZ + AmED	8	I.P. or Gavage, Chronic (14 days) PTZ was injected on days 1, 7 and 14 of treatment (for 30 min)	onset time and severity of seizures	-	EtOH and AmED decreased the onset time of the seizures after at least 7 days of treatment. EtOH and AmED equally reduced the severity of PTZ-induced seizures.	Gözler & Uzbay, 2022
Rat	Sprague-Dawley	Female	Intermittent ED and Intermittent AmED (on mondays, wednesdays and fridays), Dialy ED, Dialy AmED and Control	8	Two-bottle choice and operant self-administration, Chronic (10 weeks)	Locomotor activity and anxiety like-behavior (open field/ 5 min, 24h after administration). Memory: novel place recognition test (1 week after administration). EtOH operant self-administration occured in a FR1 schedule (20 min for 2 weeks). After, they changed to a PR schedule (for 20 minutes)	-	Intermittent AmED group had higher locomotion and less anxiety-like behavior than the Intermittent ED or the Daily ED groups. No difference was found compared with the Daily AmED. Intermittent AmED had more responses than all the other groups on the PR schedule	Williams et al., 2022

ALP= alkaline phosphatase; ALT= alanine aminotransferase; AmED= Alcohol mixed with Energy Drinks; AST= aspartate aminotransferase; BEC= Blood EtOH Concentration; FR1= fixed-ratio schedule; γ-GT= gamma-glutamyltranspeptidase; GFAP= Glial-Fibrillary-Acidic-Protein; IL-1β=interleukin 1β; i.p.= intraperitoneal; LDH= lactate dehydrogenase; PTZ= pentolenotetrazol; TBARS= Thiobarbituric acid reactive substances; TNF= proinflammatory cytokine tumor necrosis factor
